# MicroRNAs in Gingival Crevicular Fluid: An Observational Case-Control Study of Differential Expression in Periodontitis

**DOI:** 10.3390/ncrna9060073

**Published:** 2023-11-18

**Authors:** Pedro J. Almiñana-Pastor, Francisco M. Alpiste-Illueca, Pablo Micó-Martinez, Jose Luis García-Giménez, Eva García-López, Andrés López-Roldán

**Affiliations:** 1 Department of Stomatology, Faculty of Medicine and Odontology, University of Valencia, 46010 Valencia, Spain; pedro.alminana@uv.es (P.J.A.-P.); francisco.alpiste@uv.es (F.M.A.-I.); paumico90@gmail.com (P.M.-M.); andreslopezroldan@gmail.com (A.L.-R.); 2 Biomedical Research Institute INCLIVA, Consortium Center for Biomedical Network Research on Rare Diseases, CIBERER-ISCIII, 46010 Valencia, Spain; 3 Department of Physiology, Faculty of Medicine and Odontology, University of Valencia, 46010 Valencia, Spain; eva.garcia@epidisease.com

**Keywords:** microRNAs, epigenetics, periodontitis, gingival crevicular fluid

## Abstract

Objectives: microRNAs (miRNAs) present in the gingival crevicular fluid (GCF) of patients with chronic periodontitis may serve as biomarkers of periodontal disease. The aim of this study was to perform a miRNA-sequencing study of all miRNAs present in GCF, comparing miRNA expression level profiles between advanced chronic periodontitis (CP) patients and healthy subjects (HS). Materials and methods: GCF samples were collected from the single-rooted teeth of patients with severe CP (*n* = 11) and of HS (*n* = 12). miRNAs were isolated from GCF using an miRNeasy Serum/Plasma kit(Qiagen GmbH, Hilden, Germany). Reverse transcription polymerase chain reaction (qRT-PCR) was used to determine the expression levels of miRNA candidates involved in periodontal pathogenesis. Results: Of all the sequenced miRNAs, miR-199, miR-146a, miR-30a, and miR-338 were identified as best representing the CP patient samples. The validation study identified miR-199 as the most powerful biomarker used to define periodontitis. Conclusions: Upon sequencing all known miRNAs in GCF for the first time, we uncovered several potential biomarkers to define periodontitis. Identifying miRNAS in the GCF using high-throughput approaches will clarify the role of these molecules in periodontitis and provide biomarkers with potential applications.

## 1. Introduction

Periodontitis is a multifactorial inflammatory disease that compromises the integrity of the periodontium, which is formed by tissues that support the teeth such as the gingiva, periodontal ligament, dental cement, and alveolar bone [1]. In a 2016 study, Hajishengallis defined periodontitis as a chronic inflammatory disease triggered by the formation of a dysbiotic dento-gingival biofilm [2]. In periodontitis, bacterial stimuli trigger molecular signaling, which in turn initiates an immune inflammatory response by the host to halt or eliminate microorganisms. Bacteria release metabolites and enzymes that aggravate tissue damage, while leukocytes and fibroblasts produce various inflammatory mediators, including cytokines, prostaglandins, reactive oxidative species (ROS), proteolytic enzymes, and metalloproteinases [3]. 

Nevertheless, the response to bacterial stimuli can be disproportionate in some individuals, paradoxically producing a loss of tooth-supporting structure [3]. It is widely known that certain genetic disorders are associated with periodontitis. Nevertheless, not every individual with a genetic predisposition will necessarily develop periodontitis, and conversely, not every patient with periodontal problems will present these genetic disorders [4].

Other environmental factors, such as smoking and diet, play an important role in the typical oral dysbiosis of periodontal disease. They can help create a microenvironment conducive to the multiplication and survival of certain periodontal pathogens bacteria, and they can inhibit the growth of other microorganisms [5,6]. Furthermore, a significant body of evidence now supports independent associations between severe periodontitis and several systemic diseases, including diabetes, cardiovascular disease, chronic obstructive pulmonary disease, and chronic kidney disease [7,8,9,10].

MicroRNAs (miRNAs) are a group of non-coding RNAs that comprise approximately 17–25 nucleotides. They can bind complementary sequences of mRNA to post-transcriptionally regulate mRNA expression. Under certain conditions, miRNAs can therefore repress, activate, or regulate the initiation of protein translation and synthesis [11,12,13,14,15,16]. Notably, a single miRNA can potentially control the expression of hundreds of target genes simultaneously, which can substantially influence regulatory pathways such as bone metabolism and bacterial cell invasion and therefore contribute to disease pathogenesis [3,12,13]. miRNAs have been associated with chronic inflammatory processes, cancer, and other pathologies. They are found in different tissues and fluids of the body. Owing to these factors and their high stability in biological fluids, miRNAs are considered to be potential biomarkers in different clinical settings [15]. In summary, miRNAs may represent potential tools to study chronic inflammatory diseases in which environmental risk factors have a major impact on disease initiation and progression [17,18,19]. 

In periodontitis, epigenetic mechanisms can be considered reliable biomarkers that are involved in regulating adaptation to chronic inflammatory stimuli [20,21]. Several previous studies have reported significant differences in miRNA expression levels between tissues with periodontitis and healthy tissues [22,23,24,25,26,27]. However, most studies have analyzed these markers in gingival biopsies, an invasive method which reduces its value as a biomarker. Conversely, gingival crevicular fluid (GCF) represents an ideal, accessible, and non-invasive medium for studying the expression of these miRNAs [25,28,29,30]. Gingival crevice fluid (GCF) is a complex mixture of substances derived from serum, leukocytes, structural cells of the periodontium, and oral bacteria. Importantly, GCF possess great potential as an indicator of periodontal disease and healing after therapy [31]. Therefore, analysis of the circulating miRNAs present in GCF has the potential to improve the study of periodontal pathogenesis, to enhance our understanding of how environment and lifestyle can contribute to periodontitis, and also to deepen insights into its bidirectional relationship with other systemic diseases such as diabetes [32,33] and cardiovascular diseases [34,35], in which the causative relations remain elusive. 

The objective of this study was to perform small RNA sequencing (small RNA-seq) to analyze all the miRNAs present in GCF derived from patients with advanced chronic periodontitis and to compare the miRNA expression levels with those in healthy subjects. We also aimed to identify periodontitis-associated miRNAs from different biological pathways which could define periodontitis pathogenesis.

## 2. Results

### 2.1. Description of Patients

The subjects comprised a test group (CP patients, *n* = 11) and control group (HS, *n* = 12). The percentage of men in the healthy group was 33.3%, and for the periodontitis group, it was 27.3%. The percentage of women was 66.7% in the healthy group and 72.7% in the periodontitis group. In the healthy group, 75% of the subjects were non-smokers and 25% smoked less than 10 cig/day. In the periodontitis group, 63.6% were non-smokers and 36.4% smoked less than 10 cig/day. The mean participant age was 50.17 years (±7.34 years) in the test group and 46.36 years (±9.88 years) in the control group. No significant differences were found for any of the demographic parameters (sex (*p* = 0.765), age (*p* = 0.304), smoking habit (*p* = 0.406)), indicating that the groups were homogeneous (see Supplementary Materials). The study power, calculated using a post hoc analysis, was found to be 76% for this sample. The clinical variables clearly defined both groups. Significant differences (*p* < 0.0001) were found for the clinical variables (Table 1). The volume of CSF was also higher in the test group than in the control group (*p* < 0.0001).

### 2.2. miRNA Expression Analysis in Patients with Advanced Chronic Periodontitis

**Figure 1:**
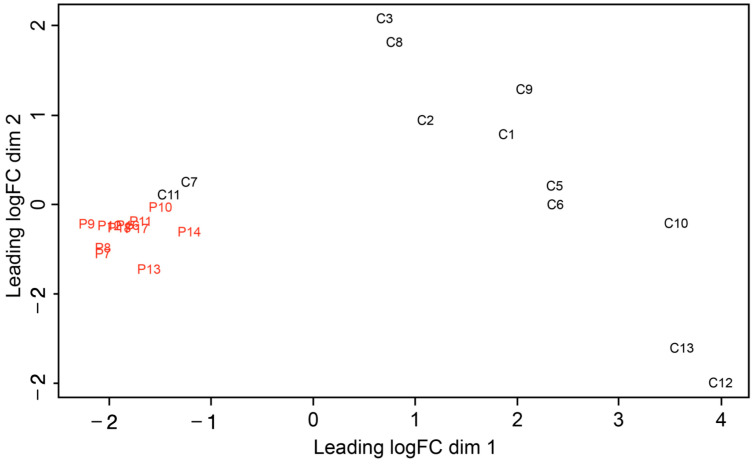
Principal component analysis of the samples. The plot shows the microRNA expression level trends between the patients and controls.

The first exploratory analysis of the small RNA-seq data was performed using a multidimensional principal component analysis evaluating the miRNA expression levels in all the samples (Figure 1).

The miRNA expression profiles revealed two clearly differentiated groups. In the test group, miRNA expression presented a homogeneous profile that was distinctly grouped in the left region of the graph, while it was more heterogeneous in healthy subjects, yet clearly different from that of the patients with periodontitis. Notably, controls 7 and 11 were positioned closer to the test group. 

**Figure 2:**
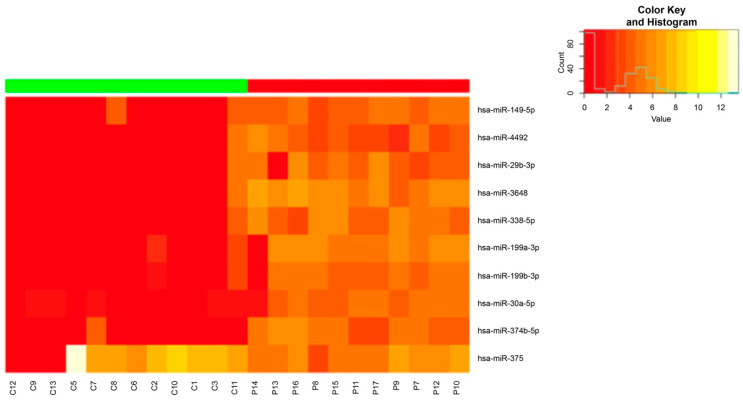
Heatmap representing microRNA expression levels. The red shades indicate low expression and predominate the control group, and the orange shades indicate greater expression of miRNAs in the test group than the control group.

A bioinformatics analysis identified 148 miRNAs showing significant differential expression between the two groups. These were selected for further analysis. The heatmap (Figure 2) revealed miRNAs whose expression clearly defined subjects in the test group. These 10 miRNAs defined a signature clearly that differentiated between CP patients and healthy subjects; therefore, they were selected as candidates for periodontitis biomarkers. 

The HS and advanced CP patients had different miRNA expression levels in the GCF. The following 10 miRNAs defined the subjects with periodontitis: hsa-miR-199a-3p, hsa-miR-199b-3p, hsa-miR-338-5p, hsa-miR-374b-5p, hsa-miR-30a-5p, hsa-miR-375, hsa-miR-3648, hsa-miR-29b-3p, hsa-miR-4492, and hsa-miR-149-5p. 

### 2.3. Selection of Reliable miRNAs to Define Periodontitis and Its Etiopathogenesis

**Figure 3:**
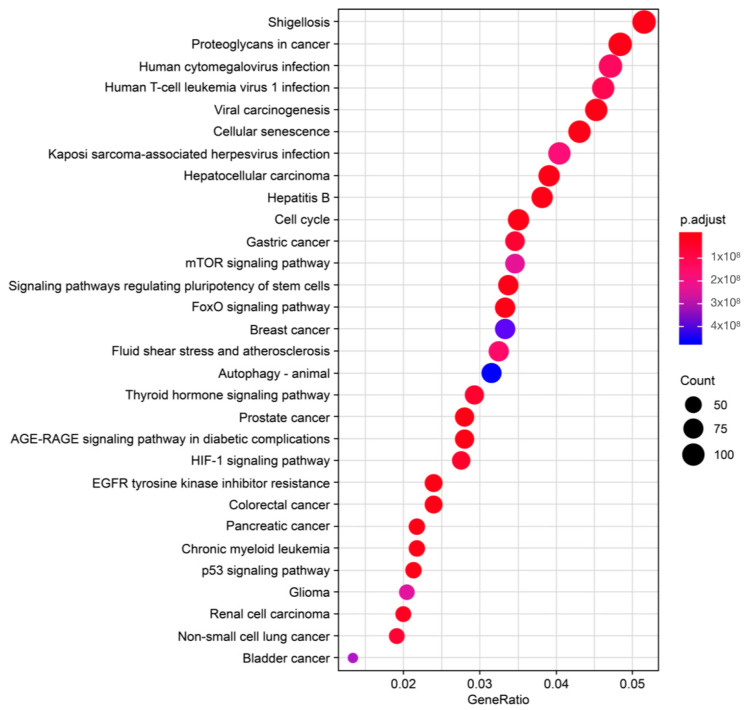
Overrepresentation analysis. The plot shows the importance of the pathways in genes with a demonstrated involvement in periodontitis.

The 10 miRNAs were analyzed using the DIANA-miRPath web tool, which allowed us to identify the target genes of each miRNA and analyze their involvement in biological KEGG pathways. These biological pathways were then manually searched in PubMed to pinpoint their direct and indirect relationships with periodontitis. Using this process, we were able to select up to 19 potential pathways that were closely related to periodontitis. The pathways were particularly linked to bone metabolism, inflammatory response, rupture of epithelial junctions, and bacterial invasion of epithelial cells. In addition, we performed an overrepresentation analysis (ORA) using the list of significant miRNAs (false discovery rate < 0.05), a relatively less strict analysis which did not take into account the differential expression analysis statistics. The ORA demonstrated that the miRNAs with differential expression were related to pathways such as proteoglycans in cancer, viral carcinogenesis, cellular senescence, cell cycle, and forkhead box class O signaling pathways. These are pathways with genes that have been proven to be involved in periodontitis (Figure 3).

Together with the power of miRNA expression, logistic regression, and the involvement in the KEGG biological pathways related to periodontal pathogenesis, a bioinformatics analysis identified miRNA signatures comprising miR-30a-5p, miR-199b-3p, miR-338-5p, and miR146a-5p as potential biomarkers of periodontitis. For miR-146a-5p, a tendency to overexpression was observed in the patients with periodontitis. Although the differences were not significant, we included this miRNA for analysis, since previous studies have highlighted its role in periodontitis [36,37].

### 2.4. Validation of Differentially Expressed miRNAs via RT-qPCR

**Figure 4:**
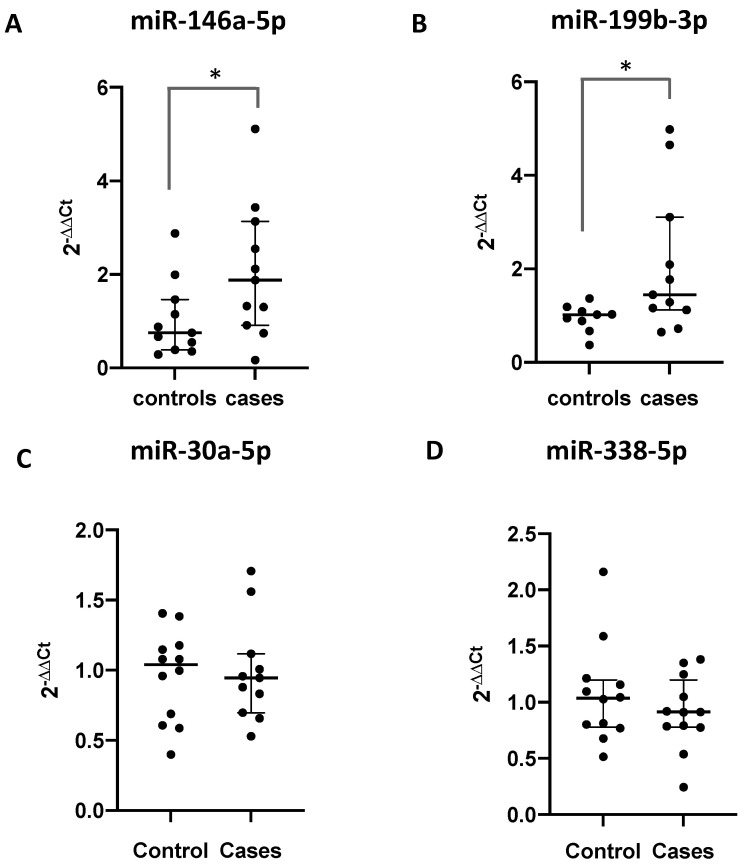
Relative expression levels of miRNAs found in the gingival crevicular fluid of patients with chronic periodontitis compared with those in healthy control participants. Box plot showing the relative expression levels of (**A**) miR-30a-5p, (**B**) miR-199a-3p, (**C**) miR-338-5p, and (**D**) miR-146a-5p in the healthy participants (controls, *n* = 12) and CP patients (cases, *n* = 11). The miRNA expression levels were normalized to those of miR-223-3p. miR-146a-5p and miR-199b-3p were overexpressed in the GCF of the periodontitis patients compared with the levels in the healthy subjects (*p* = 0.05 and *p* = 0.03, Mann–Whitney tests, respectively). The lines within the dispersion of points indicate the median. The bottom and top edges of the graph indicate the interquartile range. The symbol * indicates significant differences.

The miRNA signature proposed as a biomarker for periodontitis was validated via RT-PCR using CSF derived from different teeth obtained from the same subjects used in the small RNA-seq analysis. miR-146a-5p and miR-199b-3p were overexpressed in the GCF of the patients with periodontitis compared to their levels in the healthy subjects (*p* = 0.05 and *p* = 0.03, Mann–Whitney tests, respectively). Conversely, miR-30-5pa and miR-338-5p showed similar levels in both groups (Figure 4).

**Figure 5:**
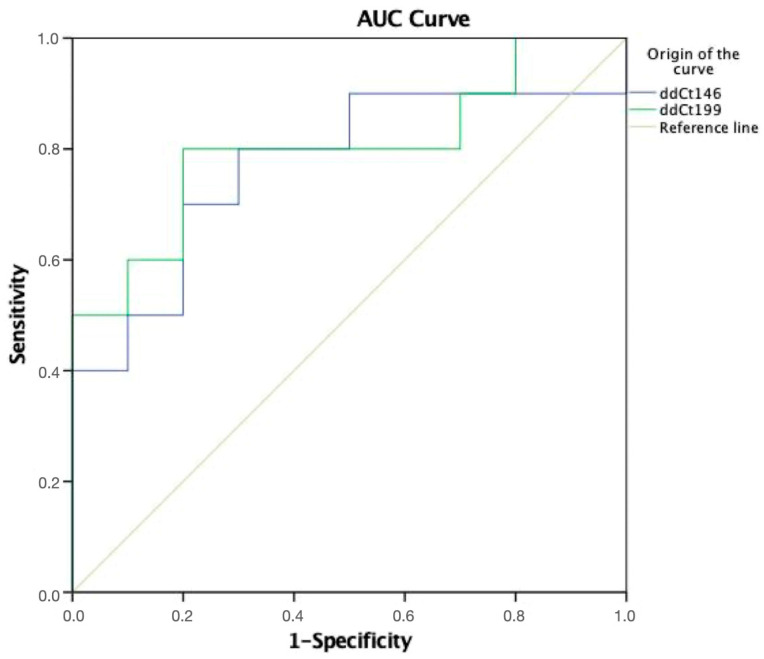
ROC curve analysis for miR-146a-5p and miR-199b-3p as diagnosis biomarkers of periodontitis. A detailed summary of the AUC, 95% CI lower and upper limit, sensitivity, and specificity of the two miRNAs are shown in the inserted Table 2. The AUCs for circulating miR-146a-5p and miR-199b-3p in the GCF were 0.74 and 0.78, respectively, indicating a high diagnostic value.

To evaluate the potential of the two validated miRNAs (miR-146a-5p and miR-199b-3) as diagnostic biomarkers, corresponding ROC curves were generated and analyzed (Figure 5). miR-199b was identified as the most powerful diagnostic biomarker of periodontitis. Improved AUC, sensitivity, and specificity values were obtained for the assay based on the use of miRNAs in GCF for the diagnosis of periodontitis. After repeating the experiment using different samples, miR146a-5p and miR-199b-3p were validated as reliable biomarkers to define advanced chronic periodontitis in GCF.

**Table 2:** Receiver operating characteristic curve analysis.

miRNA	AUC	95% CI	*p* Value	Cut-Off Value	Sensitivity	Specificity
146a-5p	0.74	0.53–0.96	0.05	1.22	72.7	72.7
199b-3p	0.78	0.57–0.99	0.09	1.11	80.0	72.7

AUC, area under the curve; CI, confidence interval.

### 2.5. Analysis of the Correlation between GCF Volume and Expression Levels of the Validated miRNAs

A Spearman’s correlation analysis was performed between the miRNA expression levels and GCF volume, which has been previously proposed as a biomarker of periodontitis. A positive correlation was found between the GCF volume and the miR-199b and miR-146a expression levels (*p* < 0.004 and *p* < 0.027), in which the expression levels of the two miRNAs increased as the GCF volume increased (Table 3).

## 3. Discussion

Biomarkers represent a useful tool to help address the challenges of implementing preventive, predictive, and personalized medicine in all healthcare disciplines. In periodontal disease, the most important challenges are to diagnose the condition before it becomes clinically detectable and to avoid progression, improve patient management, and provide treatment. In this context, miRNAs have demonstrated an extraordinary performance as biomarkers for the early diagnosis and prognosis of several diseases [15]. To the best of our knowledge, our is the first study to analyze all the miRNAs present in the GCF of patients with chronic periodontitis, performed using small RNA-seq analysis. The cohort of subjects participating in this study was homogeneous in terms of demographic variables, and the clinical parameters clearly differentiated the two groups. The GCF volume increased and changed qualitatively with inflammation; however, although GCF reflects subclinical inflammation, it is not possible to determine the cellular origin of the markers studied [38].

In this study, we analyzed the differential expression profiles of miRNAs in GCF obtained from patients with chronic periodontitis using a strategy based on small RNA-seq. The miRNA expression profile clearly differentiated CP patients with chronic periodontitis from HS, providing a potential biomarker signature to identify patients with this pathology. 

Since the sample size could not be calculated, we considered previous studies using a similar methodology for the sample size selection. In total, 23 subjects participated in our study, including 11 with advanced periodontitis and 12 healthy subjects. The power of the study was calculated via a post hoc analysis, showing a power of 76%. For future studies, 48 subjects are needed to reach 90% power. Nonetheless, our sample size can be considered sufficient, since it yielded significant differences. In our analysis, we found a signature of 10 miRNAs that could differentiate the patients from the healthy subjects. This miRNA signature was used to identify target genes which can alter the function of biological pathways related to periodontitis. Using the DIANA-miRPath web tool, we found that the selected miRNAs participated in processes related to bone metabolism, inflammatory response, rupture of epithelial junctions, and bacterial invasion of epithelial cells. The study of these molecular pathways, together with ORA, facilitated the selection of a series of miRNAs with a plausible role in periodontal pathogenesis, including miRNAs miR-30a-5p, miR-199b-3p, miR-338-5p, and miR-146a-5p. In addition, we performed a validation analysis of these miRNAs via RT-qPCR, finding that miR-146a-5p and miR-199b-3p were upregulated in the CP patients compared to the HS. Notably, an ROC curve analysis revealed miR-199b-3p to be the most powerful biomarker in periodontitis in the CP patient cohort. Moreover, among the miRNAs evaluated, miR-199b-3p and miR-146-5p showed a positive correlation with GCF volume (*p* < 0.004 and *p* < 0.027), confirming the potential use of both miRNAs as biomarkers of chronic periodontitis. 

MiR-199b-3p and miR-146a-5p have been previously associated with inflammatory diseases, cardiovascular diseases, and cancer [39]. Both miRNAs target genes such as tumor necrosis factor alpha, prostaglandin, interleukin-1 beta, interleukin-6, bone morphogenetic protein 2, epidermal growth factor, and transforming growth factor beta [37,40,41]. Moreover, they are both involved in activating nuclear factor-kappa beta and altering osteoclastogenesis (RANK/RANKL/OPG axis). The activator of the nuclear factor-κB (RANK) receptor is a transmembrane protein that is expressed in both mature osteoclasts and their progenitors, and binding to its ligand (RANKL) determines osteoclast differentiation and activation. The levels of these markers correlate with periodontitis progression [37,41,42].

Of the few studies analyzing miRNAs in GCF, none have used high-throughput screening via small RNA-seq of all miRNAs. Saito et al. employed a ready-to-use PCR with 720 miRNAs, while Radovic et al. analyzed the expression levels of two miRNAs before and after periodontal treatment in patients with periodontitis and diabetes, reporting that the expression levels of miR-146a-5p and miR-155 decreased after treatment [36,43]. Some authors have analyzed the expression level of several miRNAs in GCF, but none have carried out a complete screening of all the miRNAs [44,45,46,47,48]. The results of this study agree with the results of Rovas et al., 2022 and Zhu and Zhong, 2022, reporting high expression level of miRNAs miR-30a and miR-146a in periodontitis [45,47]. 

Regarding the two healthy subjects with overexpression of the selected periodontitis-defining miRNAs, it is feasible that these miRNAs potentially indicate an increased risk or a subclinical state of periodontitis in subjects without clinical signs of disease. Further studies and patient follow-ups are needed to confirm this hypothesis.

The study of miRNAs in a non-invasive medium like GCF has the potential to improve the use of these biomarkers, and this study demonstrates the importance of research with these miRNAs in the field of periodontology. Nonetheless, our results should be interpreted in the context of certain limitations concerning the methodology and the source used to purify miRNAs, as no previous literature exists outlining optimized procedures for smallRNA-seq in microRNAs purified from GCF. Indeed, as far as we know, this is the first study using a smallRNA-seq approach to identify potential miRNAs in periodontitis-related GCF; a significant fact, given that epigenetic biomarkers could be useful in diagnosing stage I of periodontitis, as suggested by Tonetti et al. in their new classification of periodontal diseases [49]. Another limitation of our study was the selection process for the appropriate miRNA to be used as a reference to perform the qPCR experiments. Selecting appropriate reference genes to normalize the relative miRNA expression in biofluids is a current challenge in other biospecimens such as blood and saliva, and this was also a challenge in this study using GCF. An interesting future line of research would be to analyze the value of these epigenetic biomarkers for studying the bidirectional relationship of periodontal diseases with several systemic and non-communicable diseases.

## 4. Materials and Methods

This study was conducted in compliance with the revised Declaration of Helsinki and was approved by the Experimental Research Ethics Committee of the University of Valencia, Valencia, Spain (procedure number: H1477063568528). All individuals participating in the study were informed about the objective and methods of the study and provided written informed consent. At the time of this study, no previous reports allowed the calculation of the sample size, therefore studies with a similar methodology were consulted [43,50]. We selected a group of subjects with advanced chronic periodontitis (CP) with probing depths ≥ 6 mm and dental bone loss ≥ 50% and a group of healthy subjects (HS) without periodontal pockets, dental bone loss, or gingival inflammation. Both groups had an age range of 30–60 years, comprising individuals who smoked ≤10 cigarettes/d. The exclusion criteria included patients with systemic diseases, patients under treatment or medication that could affect bone metabolism or inflammation response, and patients taking antibiotics, smoking >10 cigarettes a day, or using antiseptic mouthwashes. 

### 4.1. Clinical Records

A complete periodontal examination was performed in the periodontics clinic of the University of Valencia, which determined probing depths using a pressure-controlled probe, plaque index (plaque scoring according to Quigley and Hein, modified by Turesky et al., 1970) [51], gingival index (Ainamo and Bay, modified by Lindhe 1983) [52], clinical attachment level, bleeding on probing, furcation involvement, and mobility. In addition, complete periodontal X-rays were performed.

### 4.2. GCF Sample Collection and Small RNA Extraction and Quantification

For GCF sampling, single-rooted teeth with probing depths of ≥6 mm were selected. Three samples were taken from three different teeth in each subject. A single sample of one tooth was used for RNA sequencing, and a sample of a different tooth was used for the validation test. The remaining samples were stored at −80 °C. 

Before the sample collection, partial isolation was performed using cotton wool. The supragingival plaque was removed, and the teeth were gently air-dried in an apico-coronal direction. Next, an absorbing paper strip (Periopaper^®^; Oraflow, New York, NY, USA) was placed on a Periotron^®^ to define the zero value in the device. The same Periopaper^®^ was carefully introduced into the gingival sulcus/periodontal pocket until resistance was felt, and it was then held in place for 30 s. Finally, the Periopaper^®^ was reintroduced into the Periotron^®^ to record the periotron units, which were later converted to microliter volume units of GCF. The Periopapers^®^ were stored in 1.5 mL tubes at −80 °C for subsequent analysis in the laboratory [38]. In the control group, sample areas were selected in the same way as the test group, but the probing depth was always ≤3 mm and with no bleeding. 

Next, we proceeded to purify the miRNAs from the crevicular fluid (CSF) samples, as previously described [50]. The Periopapers^®^ were incubated in phosphate-buffered saline (pH 7) for 30 min at room temperature (22–26 °C) and centrifuged at 500 rpm (16,000× *g*) for 10 min. Subsequently, the total RNA was extracted from 200 µL of phosphate-buffered saline using an miRNeasy Serum/Plasma kit (Qiagen GmbH, Hilden, Germany) following the manufacturer’s instructions. Finally, the RNA (including miRNAs) was eluted from purification columns using 25 µL of RNAse-free water. The purified RNA (with miRNA) concentration was quantified using a NanoDrop ND 2000 UV-spectrophotometer (Thermo Fisher Scientific, Wilmington, DE, USA), and the sample quality was measured using a Small RNA Assay (NETFLEX^®^ Small RNA-Seq kit v3 for Illumina platforms; Bioo Scientific^®^ Corporation, Austin, TX, USA) and an Agilent 2100 Bioanalyzer (Agilent Technologies, Santa Clara, CA, USA).

### 4.3. Small RNA-Seq and Analysis

#### 4.3.1. Library Preparation and Next-Generation Sequencing

Small RNA libraries were generated and indexed using NEXTFLEX^®^ Small RNA-Seq kit v3 for Illumina Platforms (Bio Scientific^®^ Corporation, Perkin Elmer, Inc., Waltham, MA, USA EEUU). In this modified protocol, the libraries were selected based on size (range, 90–170 bp) using a Blue Pippin instrument (Sage Science, Beverly, MA, USA). A positive RNA control was included (Human Brain Total RNA catalog #AM7962, ThermoFisher Scientific, Waltham, MA, USA). Single-end sequencing was performed using an Illumina NextSeq platform on High-Output 1 × 50 bp Run (NextSeq 500/550 High-Output v2 75 cycles kit, FC-404-2005) (Illumina, San Diego, CA, USA). Positive RNA from the brain is often used as control in smallRNA-seq analysis because it is known to contain a mixture of high-quality smallRNAs, including miRNAs, which can help ensure that the library construction, smallRNA-seq, and bioinformatic pipelines processes are properly executed to identify the miRNAs in biological samples. 

#### 4.3.2. Differential Expression Analysis between Advanced Chronic Periodontitis Patients and Healthy Subjects

The quality of the Illumina raw sequences was assessed using FastQC software V 0.11.9 (https://www.bioinformatics.babraham.ac.uk/projects/fastqc/, accessed on 3 March 2020). Based on the results obtained, the sequence reads were trimmed to remove sequencing adapters and low-quality bases using Cutadapt software V 2.9 (http://cutadapt.readthedocs.org/en/stable/, accessed on 19 March 2020). Data of sufficient quality were mapped against the human GRCh38 reference sequence from UCSC (University of California, Santa Cruz), obtained from Ensembl. Subsequently, an intersection was performed between the aligned position of reads and the miRNA coordinates taken from miRBase v21. The alignment and quantification steps were performed using the Subread and Rsubread packages [53,54].

miRNAs with low counts were filtered out prior to further analysis, after which the trimmed mean of M-values normalization method [55] was performed to eliminate composition biases between libraries. We also estimated the specific dispersions per gene with a negative binomial distribution [56,57]. A differential expression analysis was performed by comparing miRNA expression levels between CP patients and HS using scripts DESeq [58]. Additionally, the differential expression was re-analyzed, adjusting for sex using a quasi-likelihood F test [59]. The counts obtained from sequencing were transformed via a variance stabilization method using the R DESeq package [58]. The transformed data were used to fit a logistic regression model with Lasso penalty [60] using the 10 differentially expressed miRNAs obtained by adjusting for sex and evaluating the 23 study samples as observations. 

The most important miRNAs in the model were selected by performing a cross-validation, choosing the miRNAs with coefficients different from 0 for the lambda value, with a minimum average cross-validation error. The data from the bioinformatics analysis were used to redraw a new heatmap using the 10 miRNAs finally selected to define advanced CP patients in the study.

### 4.4. Prediction of miRNA Targets and Overrepresentation Analysis (ORA)

To identify the most important pathways and genes regulated by differentially expressed miRNAs, we analyzed the overrepresented gene ontology (GO) terms and pathways using cluster Profiler. Ontology and pathway GO terms with an adjusted *p*-value < 0.05 were considered significantly overrepresented. We also used the DIANA-miRPath v3.0 functional analysis online suite to identify miRNAs controlling significant molecular pathways annotated in the Kyoto Encyclopedia of Genes and Genomes (KEGG) using the following default parameters: base v.7.0, a *p* value threshold of 0.001, and a microT threshold of 0.8. To reduce the number of false-positive miRNA targets, we applied a false discovery rate correction to the selected KEGG pathways. The algorithm used in this analysis was a one-tailed Fisher exact test [61]. We analyzed the pathways that have been previously associated with periodontal diseases. Finally, we chose the pathways and genes that were shared between GO and KEGG.

### 4.5. miRNA Validation Using Quantitative Reverse Transcription Polymerase Chain Reaction (RT-qPCR) in CSF Samples

RT-qPCR was performed to evaluate the eight miRNAs identified via the small RNA-seq analysis. A total of 23 GCF samples (11 from CP patients and 12 from HS) were analyzed. These 23 samples comprised different samples from a different tooth of each subject used in the small RNA sequencing analysis. RT-PCR was performed using the TaqMan^®^ miRNA Reverse Transcription kit (Part No. 4366596; Applied Biosystems, Carlsbad, CA, USA), miRNA-specific stem-loop primers (Part No. 4366596; Applied Biosystems) (hsa-miR-338-5p (assay ID 002658, ThermoFisher Scientific), hsa-miR-199a-3p (assay ID 002304, ThermoFisher Scientific), hsa-miR-30a-5p (assay ID 000417, ThermoFisher Scientific), and hsa-miR-146a-5p (assay ID 000468; ThermoFisher Scientific)), and 100 ng of input cell-free RNA in a 20 µL RT reaction. A differential expression analysis showed that miR-223-3p had the most stable and homogeneous counts among the samples; therefore, this miRNA was used as an endogenous reference control (assay ID 002295, ThermoFisher Scientific). The miRNA expression levels were normalized to the level of has-miR-233-3p, which was used as a control. 

The RT-PCR conditions were as follows: 16 °C for 30 min, followed by 42 °C for 30 min and a final inactivation step at 85 °C for 5 min. The real-time PCR reactions were performed in triplicate in scaled-down 10 µL reaction volumes using 5 µL of TaqMan^®^ 2× Universal PCR Master Mix with No UNG (Applied Biosystems), 0.5 µL of TaqMan^®^ Small RNA assay (20×) (Applied Biosystems), 3.5 µL of nuclease-free water, and 1 µL of RT product. The PCR conditions were as follows: 95 °C for 10 min, followed by 45 cycles at 95 °C for 15 s and 60 °C for 1 min. As above, hsa-miR-223-3p miRNA served as an endogenous control for normalization. The relative quantification of miRNAs was calculated using the 2^(−ΔΔCT)^ method [62].

### 4.6. Statistical Analysis

The mean was taken as the best measure of tendency, and the standard deviation (SD) was taken as the measure of dispersion. In a descriptive analysis of the sample, the corresponding frequency tables were constructed for the qualitative variables, while descriptive statistics [mean, median, confidence interval (CI) for the mean, and standard deviation (SD)] were calculated for the quantitative variables. For comparison of means, normality tests were always applied with the objective of evaluating the appropriate comparison test (Student’s *t* test or Mann–Whitney U test). For comparisons of categorical variables such as sex and smoking habits, Chi-squared tests and Fisher’s exact tests were performed. 

For validation analysis, the Mann–Whitney test was used to change the miRNA values to continuous values to allow between-group comparisons. For multiple comparisons, Kruskal–Wallis and Dunns post hoc tests were applied. The diagnostic capacity of each miRNA was validated using ROC (Receiver Operating Characteristic) curve analysis, calculating the area under the curve, the sensitivity and specificity, the positive predictive value (PPV), and the negative predictive value (NPV). The optimal cut-off point was determined by high values of sensitivity, specificity, and efficiency using the Youden index. Statistically significant differences were assumed when the *p*-value was less than 0.005(*). SPSS (IBM^®^ SPSS^®^ statistics version 24.0) was used for the calculations.

## 5. Conclusions

Notwithstanding the limitations of our study, our findings provide an miRNA signature comprising miR-199b-3p and miR-146a-5p that defines periodontitis, thus warranting future studies on this biomarker signature to evaluate its performance for identifying patients with a subclinical phenotype. Furthermore, it would be useful to identify biomarkers to distinguish different patient subsets and prevent the appearance of sequelae in periodontitis. These multiple applications of miRNA-based biomarkers in GCF could improve our knowledge of the relationship between periodontitis and systemic diseases and may help to implement precision periodontics.

## Figures and Tables

**Table 1:** Variables evaluated in the patient and control groups.

Variable	Healthy Subjects	CP Patients	*p* Value
CAL	2.92 (CI 2.66–3.17) (SD ± 0.40)	5.06 (CI 4.60–5.52) (SD ± 0.68)	*p* < 0.0001
Mean PD	2.29 (CI 2.14–2.44) (SD ± 0.23)	4.04 (CI 3.76–4.31) (SD ± 0.41)	*p* < 0.0001
Mean PI	2.03 (CI 1.70–2.36) (SD ± 0.52)	3.32 (CI 2.85–3.61) (SD ± 0.56)	*p* < 0.0001
BOP percentage	13.90% (CI 5.09–22.72) (SD ± 13.87)	80.19% (CI 67.5–92.8) (SD ± 18.83)	*p* < 0.0001

CAL, clinical attachment level; PD, probing depth; PI, plaque index; BOP, bleeding on probing; CI, confidence interval; CP, chronic periodontitis; SD, standard deviation. *t* tests were used to the CAL, PD, PI, and BOP.

**Table 3:** Analysis of the correlation between the GCF volume and the expression levels of the validated miRNAs. Rho Sperman’s test was used.

			GCF Volume Tooth 1	GCF Volume Tooth 2	GCF Volume Tooth 3
**Rho Spearman**	**2-^ΔΔCt^** **miR-146a-5p**	**Correlation coefficient**	0.483 *	0.514 *	0.561 **
		**Sig. (bilateral)**	**0.027**	**0.017**	**0.008**
		***n***	21	21	21
	**2-^ΔΔCt^** **miR-199b-3p**	**Correlation coefficient**	0.615 **	0.686 **	0.717 **
		**Sig. (bilateral)**	**0.004**	**0.001**	**0.000**
		***n***	20	20	20
	**2-^ΔΔCt^** **miR-30a-5p**	**Correlation coefficient**	0.114	0.116	0.166
		**Sig. (bilateral)**	0.624	0.616	0.471
		***n***	21	21	21
	**2-^ΔΔCt^** **miR-338-5p**	**Correlation coefficient**	−0.001	0.024	−0.072
		**Sig. (bilateral)**	0.996	0.914	0.744
		***n***	23	23	23

* and ** indicates significant differences. *: Significant correlation at level 0.05 (bilateral). **: Significant correlation at level 0.01 (bilateral).

## Data Availability

The data that support the findings of this study are available from the corresponding author upon reasonable request.

## Supplementary Materials

The following supporting information can be downloaded at: https://www.mdpi.com/article/10.3390/ncrna9060073/s1, Table S1: Descriptive analysis of the sample: tobacco and age; Table S2: Descriptive analysis of the sample: sex; Table S3: Descriptive analysis of the sample: cigarrets/day; Table S4: Descriptive analysis of the sample: Age; Table S5. Independent sample test: Age.
