# Supplementary material for: MicroRNAs in Gingival Crevicular Fluid: An Observational Case-Control Study of Differential Expression in Periodontitis
Source: Noncoding RNA. 2023 Nov 18;9(6):73. doi: 10.3390/ncrna9060073 (PMC10660715; doi:10.3390/ncrna9060073)
Supplement: Supplementary file 1 [file ncrna-09-00073-s001.zip › ncrna-2650751-supplementary.pdf]

Supplementary Materials

Table S1. Descriptive analysis of the sample: tobacco and age.

	Group			Estadístico	Error estándar
Number cigar-rets/day	control	Mean		1.42	0.763
		95% Confidence Interval for the mean	Lower limit	−0.26	
			Upper limit	3.10	
		Mean cropped at 5%		1.19	
		Median		0.00	
		Variance		6.992	
		Standard deviation		2.644	
		Minimum		0	
		Maximum		7	
		Range		7	
		Interquartile range		3	
		Asymmetry		1.546	0.637
		Kurtosis		0.781	1.232
	case	Mean		2.91	1.282
		95% confidence Interval for the mean	Lower limit	0.05	
			Upper limit	5.77	
		Mean cropped at 5%		2.68	
		Median		0.00	
		Variance		18.091	
		Standard deviation		4.253	
		Minimum		0	
		Maximum		10	
		Range		10	
		Interquartile range		7	
		Asymetry		0.982	0.661
		Kurtosis		−0.886	1.279
AGE	control	Mean		50.17	2.121
		95% Confidence Interval for the mean	Lower limit	45.50	
			Upper limit	54.83	
		Mean cropped at 5%		50.46	
		Median		50.50	
		Variance		53.970	
		Standard deviation		7.346	
		Minimum		35	
		Maximum		60	

		Range		25	
		Interquartile range		10	
		Asymetry		-0.651	0.637
		Kurtosis		0.212	1.232
	case	Mean		46.36	2.980
		95% Confidence Interval for the mean	Lower limit	39.72	
			Upper limit	53.00	
		Mean cropped at 5%		46.07	
		Median		44.00	
		Variance		97.655	
		Standard deviation		9.882	
		Minimum		35	
		Maximum		63	
		Range		28	
		Interquartile Range		18	
		Asymetry		0.354	0.661
		Kurtosis		−1.193	1.279

Table S2. Descriptive analysis of the sample: sex.

		Frequency	Percentage	Valid Percentage	Cumulative Percentage
	Man	4	33.3	33.3	33.3
	Women	8	66.7	66.7	100.0
	Total	12	100.0	100.0	

a. Control

		Frequency	Percentage	Valid Percentage	Cumulative Percentage
Válido	man	3	27.3	27.3	27.3
	women	8	72.7	72.7	100.0
	Total	11	100.0	100.0	

a. Case

Table S3. Descriptive analysis of the sample: cigarrets/day.

	Frequency	Percentage	Valid Percentage	Cumulative Percentage
0	7	63.6	63.6	63.6
5	1	9.1	9.1	72.7
7	1	9.1	9.1	81.8
10	2	18.2	18.2	100.0
Total	11	100.0	100.0	

a. Case

	Frequency	Percentage	Valid Percentage	Cumulative Percentage
0	9	75.0	75.0	75.0
4	1	8.3	8.3	83.3
6	1	8.3	8.3	91.7
7	1	8.3	8.3	100.0
Total	12	100,0	100.0	

a. control

Table S4. Descriptive analysis of the sample: Age.

	Group	N	Mean	Standard deviation	Standard error mean
AGE	control	12	50.17	7.346	2.121
	case	11	46.36	9.882	2.980

Table S5. Independent sample test: Age.

		Levene´s test for equality of variances		T test for equality of means						
									95% confidence interval of the mean	
		F	Sig.	t	gl	Sig. (bilateral)	Mean difference	Standard error difference	Lower	upper
Age	Equal variances are assumed	2.343	0.141	1.054	21	0.304	3.803	3.609	−3.703	11.309
	Equal variances are not assumed			1.040	18.404	0.312	3.803	3.657	−3.868	11.474
